# Contouring in transition: perceptions of AI-based autocontouring by radiation oncologists and medical physicists in German-speaking countries

**DOI:** 10.1007/s00066-025-02403-1

**Published:** 2025-04-28

**Authors:** Samuel M. Vorbach, Florian Putz, Ute Ganswindt, Stefan Janssen, Maximilian Grohmann, Stefan Knippen, Felix Heinemann, Rami A. El Shafie, Jan C. Peeken

**Affiliations:** 1 Department of Radiation Oncology, Medical University of Innsbruck, Innsbruck, Austria; 2 Department of Radiation Oncology, Universitätsklinikum Erlangen, Friedrich-Alexander-Universität Erlangen-Nürnberg, Erlangen, Germany; 3 Department of Radiation Oncology, University of Lübeck, Lübeck, Germany; 4 Department of Radiotherapy and Radiation Oncology, University Medical Center Hamburg-Eppendorf, Hamburg, Germany; 5 Department of Radiation Oncology, Helios Hospitals Schwerin, Schwerin, Germany; 6 Department of Radiation Oncology, University Medical Center Freiburg, Freiburg, Germany; 7 Radiotherapy and Radiation Oncology, University Medical Center Göttingen, Göttingen, Germany; 8 Department of Radiation Oncology, Klinikum rechts der Isar, Technical University of Munich (TUM), Munich, Germany; 9 Digitalization and Artificial Intelligence Working Group, German Society for Radiation Oncology, Berlin, Germany; 10 Partner Site Munich, German Consortium for Translational Cancer Research (DKTK), Munich, Germany; 11 Institute of Radiation Medicine (IRM), Helmholtz Zentrum München (HMGU), Munich, Germany

**Keywords:** Artificial intelligence, Questionnaires, Autocontouring, Perceptions, Radiation oncology

## Abstract

**Background:**

Artificial intelligence (AI)-based autocontouring software has the potential to revolutionize radiotherapy planning. In recent years, several AI-based autocontouring solutions with many advantages have emerged; however, their clinical use raises several challenges related to implementation, quality assurance, validation, and training. The aim of this study was to investigate the current use of AI-based autocontouring software and the associated expectations and hopes of radiation oncologists and medical physicists in German-speaking countries.

**Methods:**

A digital survey consisting of 24 questions including single-choice, multiple-choice, free-response, and five-point Likert scale rankings was conducted using the online tool umfrageonline.com (enuvo GmbH, Pfäffikon SZ, Switzerland).

**Results:**

A total of 163 participants completed the survey, with approximately two thirds reporting use of AI-based autocontouring software in routine clinical practice. Of the users, 92% found the software helpful in clinical practice. More than 90% reported using AI solutions to contour organs at risk (OARs) in the brain, head and neck, thorax, abdomen, and pelvis. The majority (88.8%) reported time savings in OAR delineation, with approximately 41% estimating savings of 11–20 min per case. However, nearly half of the respondents expressed concern about the potential degradation of resident training in sectional anatomy understanding. Of respondents, 60% would welcome guidelines for implementation and use of AI-based contouring aids from their respective radiation oncology societies. Respondents’ free-text comments emphasized the need for careful monitoring and postprocessing of AI-delivered autocontours as well as concerns about overreliance on AI and its impact on the development of young physicians’ contouring and planning skills.

**Conclusion:**

Artificial intelligence-based autocontouring software shows promise for integration into radiation oncology workflows, with respondents recognizing its potential for time saving and standardization. However, successful implementation will require ongoing education and curriculum adaptation to ensure AI enhances, rather than replaces, clinical expertise.

## Introduction

The rapid development of artificial intelligence (AI) offers great promise in various aspects of healthcare, from drug discovery to cancer diagnosis [1–3]. Within the field of radiation oncology, AI has the potential to transform several aspects of the radiotherapy workflow [4, 5].

A critical step in radiation oncology is the precise graphical delineation of the target volumes to be irradiated and the organs at risk (OARs) to be spared. Errors in this process can have serious consequences: insufficient tumor contouring can lead to underdosing, which can affect tumor control and survival; conversely, overestimation of the target volume or inadequate contouring of OARs can lead to excessive radiation exposure to healthy tissue, which increases the risk of toxicity and impairs the patient’s quality of life [6, 7].

Manual contouring, traditionally performed by radiation oncologists, is time consuming [8] and subject to inter- and intraobserver variability both within and across radiotherapy centers [9–12]. Early approaches to autosegmentation mainly used conventional techniques such as intensity analysis, shape modeling, and atlas-based methods [13]. These then-innovative approaches are limited in terms of availability, accuracy, and adaptability, especially for anatomical variations and different cancer types, and still require significant manual effort [14–17].

The emergence of deep learning techniques, particularly convolutional neural networks (CNNs), has led to significant improvements in the performance of autocontouring algorithms. These multilayer feed-forward neural networks and, in particular, specialized variants based on the U‑Net architecture, are optimized for the segmentation of image datasets. Their architecture consists of a contracting and an expanding path. The encoder or contracting path in a U-Net is responsible for progressively downsampling the input image to capture high-level features. At the deepest layer, often referred to as the bottleneck, the image is represented in its most abstract form. The decoder or expansion path then gradually upsamples this representation to generate a detailed segmentation map. Skip connections between corresponding layers of the encoder and decoder allow the model to transfer spatial and contextual information from the input image, thereby preserving important macroscale details in the final segmentation map [18].

As AI-based autocontouring tools move from research projects into routine clinical use, several vendors now offer commercially available AI-based autocontouring software tools [19, 20]. Their integration into clinical routine has represented a remarkable advancement. Recent studies have shown the benefits of AI-based autocontouring software for various tumor sites: the authors highlighted the potential to increase efficiency (time savings when contouring OARs) [21, 22] as well as to reduce contouring and dose inconsistencies, thereby contributing to standardization and quality assurance [23].

However, the introduction of AI-powered autocontouring software into clinical practice also raises significant concerns about quality assurance, education and training, potential deskilling of clinicians, and overreliance on automated systems as well as perceived risks and barriers to implementation.

Few studies have attempted to capture the views of radiation oncologists on these factors. For example, Zhai et al. [24] developed and tested a model to investigate factors influencing the acceptance of AI contouring technology in China. At the same time, Hindocha et al. [25] conducted a survey among clinical oncologists in the UK in which 78% reported that AI would have a positive impact on radiation oncology. The presented study aimed to assess the perceptions of radiation oncology professionals in German-speaking countries regarding AI-based autocontouring software and its current use, making it the first survey of its kind in this region.

## Methods

To assess the current use and potential benefits and risks of AI-based autocontouring software, the survey questions were grouped into different categories. The categories included sociodemographic data, such as age, gender, or country of residence, as well as more specific data such as experience in using AI-based autocontouring software, the need for an implementation guideline, or the perception of AI-based autocontouring software as a potentially dangerous tool. All respondents first answered the questions on sociodemographic data. All participants who reported already using an AI-based autocontouring software in clinical routine answered questions about their experience with the software. Those who do not use AI-based autocontouring software solutions skipped these questions and were redirected to questions about their general perceptions and opinions on AI-based autocontouring software.

The anonymous survey included single- and multiple-choice questions, five-point Likert scale questions (scale points ranging from 1 = disagree to 5 = fully agree), and the possibility to add further comments in a free-text box (see the Appendix for the full questionnaire). The questions were selected in a multistep process by members of the Digitalization and Artificial Intelligence Working Group of the German Society for Radiation Oncology (DEGRO). The commercially available online survey tool umfrageonline.com (enuvo GmbH, Pfäffikon SZ, Switzerland) was used for this study. The corresponding survey link was sent out via the professional mailing lists of the DEGRO and the Austrian Society for Radiation Oncology (ÖGRO). The survey was open from June 11 to August 3, 2024. An initial invitation and a reminder email were sent during this period. Participation in the survey was both voluntary and anonymous, and all respondents agreed to publication of the study results. Ethical approval was not required for an anonymized questionnaire without patient data.

### Data analysis

Raw data were obtained directly from the online tool umfrageonline.com (in Excel V16; Microsoft, Redmond, WA, USA) and then exported to SPSS Statistics (V26; IBM Corporation, Armonk, NY, USA). Responses were analyzed using descriptive statistics. The Wilcoxon rank-sum test was used to compare responses on ordinal scales between the two subgroups. Nominal data were analyzed using the chi-squared test. When the expected number of observations in more than 20% of the cells was less than 5, Fisher’s exact test was used. A value of *p* < 0.05 was considered as statistically significant. To account for multiple testing in pairwise comparisons, Bonferroni correction was applied. Free-text comments were reviewed by two authors (SMV, JCP) and grouped into topic categories for further analysis.

## Results

### Study participants

The DEGRO professional mailing list contained 1440 email addresses and the ÖGRO mailing list contained 282 email addresses. After removing duplicate addresses, nonfunctional addresses, and those belonging to retired colleagues, 1568 valid email addresses were identified. Of these, 188 participated in the survey, resulting in a response rate of 12.0%. In total, 163 out of 188 (86.7%) questionnaires were completed in full. Personal characteristics of these participants are summarized in Table 1. Overall, 89.6% of the participants were physicians, of whom more than 80% were specialized in radiation oncology. Of the participants, 85 (52.2%) were female and 46.0% were between 30 and 49 years of age. The current place of employment was a university hospital, a non-university hospital, an ambulatory health center, and a medical practice for 84 (51.5%), 23 (15.3%), 34 (20.9%), and 20 (12.3%) respondents, respectively, with Germany being the primary country (67.5%) of employment.

**Table 1:** Characteristics of the participants

Characteristic	Participants
*Profession*	
Resident	28 (17.2%)
Radiation oncology specialist	118 (72.4%)
Physicist	17 (10.4%)
*Age (years)*	
20–29	7 (4.3%)
30–39	38 (23.3%)
40–49	37 (22.7%)
50–59	53 (32.5%)
≥ 60	28 (17.2%)
*Gender*	
Female	85 (52.2%)
Male	78 (47.8%)
*Country in which profession is practiced*	
Germany	110 (67.5%)
Austria	49 (30.0%)
Switzerland	4 (2.5%)
*Institution type*	
University hospital	84 (51.5%)
Non-university hospital	25 (15.3%)
Ambulatory health center	34 (20.9%)
Medical practice	20 (12.3%)

### Current use of AI-based autocontouring software

In 114 responses (69.9%), physicians are responsible for contouring the OARs, with an almost even split between residents (*n* = 54) and radiation oncology specialists (*n* = 60). While 107 respondents (65.7%) indicated that AI-based autocontouring software is already in use in clinical routine, 22.1% of respondents do not use it and do not currently plan to implement it. The proportion of respondents reporting AI-based autocontouring software use varied across institutional types: 72.6% (61 users of 84 respondents) in university hospitals, 36.0% (9 of 25) in non-university hospitals, 64.7% in ambulatory health centers (22 of 34), and 75.0% (15 of 20) in medical practices. Chi-squared test revealed a significant association between institution type and the use of AI-based autocontouring software (*p* = 0.006), with significantly more respondents of university hospitals, ambulatory health centers, and medical practices reporting its use in clinical routine (*p* < 0.001) compared to those reporting from non-university hospitals.

The top three AI-contouring-based software products used comprised Limbus AI Inc. (30.8%), three slightly differing software solutions of Siemens Healthineers (27.1%), and ART-Plan. One respondent indicated that they use Elements (Brainlab AG), which is an atlas-based and not an AI-based autocontouring software. The total list of eight AI-based autocontouring software solutions used by the 107 user respondents are listed in Table 2. More than half of them (54.2%) have been using the software for 1–3 years, while 36.5% have been using it for less than a year. In 71% of all cases, the software runs on a local server. Before purchasing an AI-based autocontouring software, half (50.5%) of the respondents had tried several products. Artificial intelligence-based autocontouring was used for OAR contouring of the brain, head and neck, thorax, abdomen, and pelvis by more than 90% of the respondents. 56.1% of the participants dispose of an AI solution that offers automatic segmentation of target volumes. However, only 40% of these users stated that it actually saves time in the contouring process. The responses regarding current utilization of AI-based autocontouring software are summarized in Table 2.

**Table 2:** State of artificial intelligence-based autocontouring software

Characteristic	
*Responsible for OAR contouring are*	
Residents	54 (33.1%)
Radiation oncology specialists	60 (36.8%)
Physicists	11 (6.8%)
Radiation therapists	38 (23.3%)
*Current use of AI-based autocontouring software*	
Clinical routine	107 (65.7%)
Research	3 (1.8%)
None, implementation planned	17 (10.4%)
None, not planned to be implemented	36 (22.1%)
AI-based autocontouring software used*	
MVision (MVision AI Oy, Helsinki, Finland)	11 (10.3%)
Limbus AI (Limbus AI Inc., Regina, SK, Canada)	33 (30.8%)
ART-Plan (TheraPanacea, Paris, France)	16 (15.0%)
AI Rad Companion Organs RT/Syngo.via/Direct Organs (Siemens Healthineers AG, Erlangen, Germany)	29 (27.1%)
Ray Station (RaySearch Laboratories AB, Stockholm, Sweden)	9 (8.4%)
Contour Protégé AI (MIM Software Inc., Cleveland, OH, USA)	2 (1.9%)
Elements (Brainlab AG, Munich, Germany)	1 (0.9%)
Name not recalled	6 (5.6%)
AI-based autocontouring software is operated*	
On a local server	76 (71.0%)
Cloud-based	31 (29.0%)
Duration of use of AI-based autocontouring* software	
< 1 year	39 (36.5%)
1–3 years	58 (54.2%)
> 3 years	10 (9.3%)
Evaluation of different products before purchase*	
Yes	54 (50.5%)
No	53 (49.5%)
For which OARs are AI-based autocontouring software used?*^,^**	
Brain	98 (91.6%)
Head and neck	100 (93.5%)
Thorax	104 (97.2%)
Abdomen	101 (94.4%)
Pelvic	102 (95.3%)
Spine	87 (81.3%)
Do you use automatically generated structures for CTV definition?*	
Not possible	47 (43.9%)
No benefit	36 (33.7%)
Saves time in the contouring process	24 (22.4%)

*OAR* organs at risk, *CTV* clinical target volume

*100% corresponds to 107 answers

**multiple answers possible

**Fig. 1:**
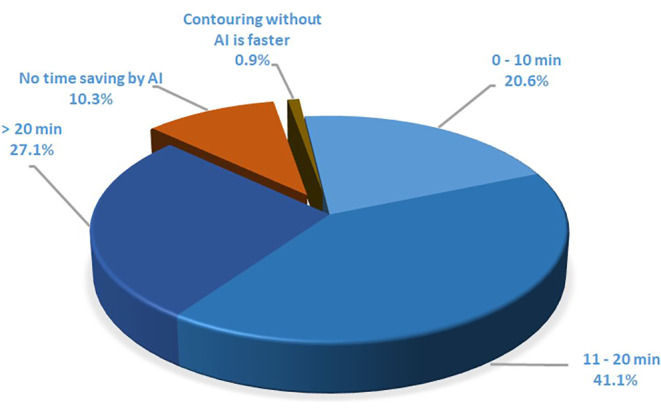
Estimation of time savings per organ at risk contouring with artificial intelligence(*AI*)-based autocontouring software

Time savings in OAR delineation were reported by 88.8% of participants, with only one person (0.9%) stating that the use of AI-based autocontouring software increased the time required to delineate OARs. The time saved using AI-based autocontouring software was estimated to be between 11 and 20 min per case for 41.1% of the respondents, while 27.1% described time savings of even more than 20 min (Fig. 1). There were no significant differences in terms of time savings depending on the profession (specialists vs. residents, *p* = 0.162; and physicians vs. physicists, *p* = 0.917) or on the type of institution (university hospitals vs. other, *p* = 0.299). Finally, there was no significant difference in the time saved during contouring depending on the type of software used (Limbus AI vs. other, *p* = 0.825; Siemens Healthineers software solutions vs. other, *p* = 0.213; and ART-Plan vs. other, *p* = 0.541).

**Fig. 2:**
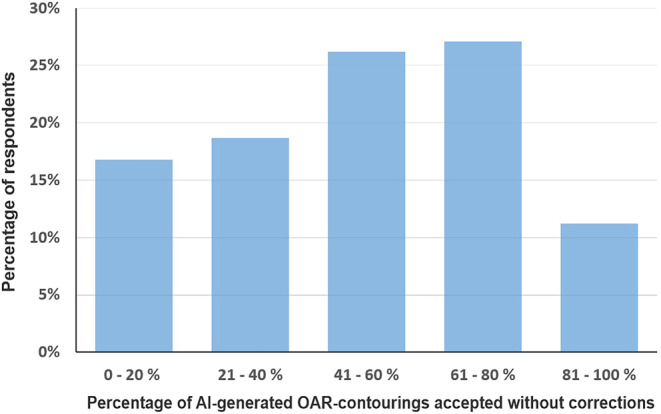
Proportion of respondents reporting different acceptance rates of artificial intelligence (*AI*)-autosegmented organs at risk (*OARs*) without manual correction

The frequency with which AI-based OAR contouring can be accepted without correction varies. For example, 16.8% of respondents stated that a correction is required in four out of five cases, while 11.2% stated that 80–100% of self-segmented OARs can be accepted without correction (Fig. 2). Around a quarter of respondents (26.2%) estimated that no correction is required in 41–60% of cases, while a further quarter (27.1%) stated that 61–80% of self-segmented OARs can be accepted without manual correction. There were no significant differences in the acceptance rate of AI-based OARs for different professional groups (specialists vs. residents, *p* = 0.111; and physicians vs. physicists, *p* = 0.989) or types of institution (university hospitals vs. other, *p* = 0.322). There were no significant differences in the acceptance rate of OARs of different AI software solutions between the respective user groups (Limbus AI vs. other, *p* = 0.142; Siemens Healthineers software solutions vs. other, *p* = 0.161; and ART-Plan vs. other, *p* = 0.426).

A minority of respondents (24.3%) stated that using their current software solution reduces the need for staff in their department. Conversely, a significant proportion (64.5%) rather disagreed or disagreed with this statement, indicating widespread skepticism about its impact on human resources. However, 92% of respondents agreed that the AI-based autocontouring software is helpful in clinical practice.

Of all respondents who use an AI solution, 87.7% do not consider it dangerous in principle. When asked whether they believe that use of an AI-based autocontouring software will lead to greater standardization and quality assurance, 79.1% of participants agreed or somewhat agreed.

**Fig. 3:**
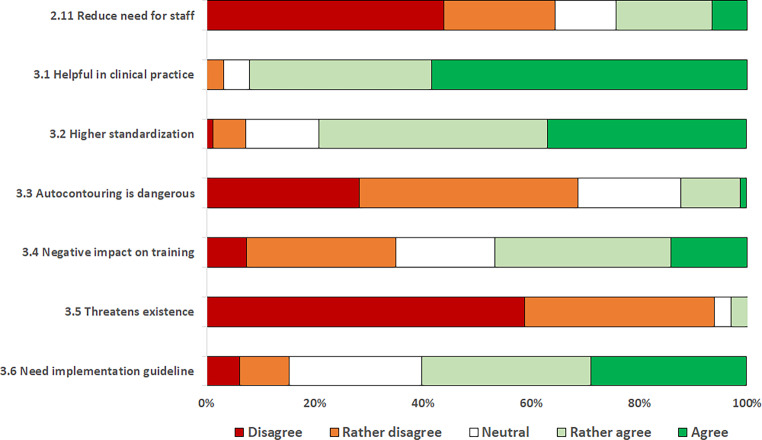
Perceptions of artificial intelligence (*AI*)-based autocontouring software based on a five-item Likert scale. For graphical purposes, the questions were paraphrased. The original questionnaire is reported in the Appendix

However, almost half (46.6%) rather agreed or agreed that the increasing use of AI-based autocontouring software will degrade resident training in understanding sectional anatomy. Even with the increasing use of AI-based autocontouring software solutions, the vast majority (93.2%) do not believe that the raison d’être of radiation oncologists will be threatened. Guidelines for the implementation and use of AI-based autocontouring software solutions provided by the respective radiation oncology societies would be welcomed by 60.1% of participants (Fig. 3).

Perceptions of the AI-based autocontouring software were homogeneous within the physician professional group: no significant difference was found between specialists and residents across any of the Likert scale responses (*p*-values ranging from 0.116 to 0.810).

Physicists, on the other hand, were more likely than physicians to agree that the use of AI-based autocontouring software will decrease the demand for radiation oncologists in the future, even if they still tend to disagree with this statement overall (median Likert scaling [LS]: 2 vs. 1, *p* = 0.037). In addition, physicists are more likely to agree that the increasing use of AI-based autocontouring tools will degrade resident training in understanding sectional anatomy compared to physicians (median LS: 4 vs. 3, *p* = 0.037).

Participants working in Germany are more likely than participants from other countries to state that the increasing use of AI-based autocontouring software will degrade resident training in understanding sectional anatomy (median LS: 4 vs. 3, *p* < 0.001).

Although agreement was high in both groups, participants working at a university hospital were more likely than those working at a non-university institution to agree that the respective radiation oncology societies should establish guidelines for the implementation and use of AI-based autocontouring software (median LS: 4 vs. 4, *p* = 0.015).

We asked respondents to comment on their views or experiences with AI-based autocontouring software in a free-text box. Free-text comments revealed several key aspects, including the need for careful oversight and manual correction of AI-based contours as well as concerns about the high cost of implementation and its potential impact on education and training. While the respondents acknowledged the potential of AI to improve efficiency and standardization, they also highlighted risks such as overreliance on automation and its potential impact on the skill development of younger clinicians. A detailed analysis of the free-text responses is provided in the Appendix.

## Discussion

This study offers insights into the perspectives, current applications, and obstacles of AI-based autocontouring software in radiation oncology across DEGRO and ÖGRO members. The results support the implementation and acceptance of AI-based autocontouring software, which is of utmost importance given the huge potential of AI in radiation oncology.

Previous studies have highlighted various aspects of AI-based autocontouring. Zhai et al. [24] reported on a self-developed model to assess the acceptance of AI-based autocontouring software in China. Among the 307 respondents, technical resistance was low, and the overall perception of AI was high. However, current usage, fears, and expectations were not captured in this study, which may be due to the fact that almost 60% of the respondents had not yet used AI-based autocontouring software and almost a quarter of the respondents were still medical students. Mugabe [26] reported the views of a multidisciplinary group including 15 radiation oncologists from New Zealand. However, they focused on the impact of AI in general, and only 35% reported using AI tools for autosegmentation. Brouwer et al. reported on the perception of 213 medical physicists from 202 radiation oncology centers across Europe regarding AI applications in general [27]. Wong et al. [28] reported on perceptions of Canadian radiation oncologists, physicists, radiation therapists, and radiation trainees regarding the general impact of AI. To date, only two surveys have been conducted that truly focus on AI-based autocontouring software: Hindocha et al. [25] reported on the responses of 51 clinical oncologists in the UK and Bourbonne et al. [20] reported on the French perspective of young radiation oncologists (85% residents).

In our survey, 65.7% of respondents reported using AI-based autocontouring software in routine clinical practice, which is higher than the 45% of respondents in the UK survey [25] and closer to the 60.7% of French respondents [20]. Like our study, these two surveys are not representative because they report on different numbers of respondents per center and did not include all centers in each country, so there are no data on the true prevalence of AI use.

Nevertheless, these studies suggest that the clinical use of AI technologies in radiation oncology is still in its early stages. A study conducted in New Zealand [26] reported that “AI usage was low” but overall, respondents had “a high likelihood to adopt AI.” Similarly, nearly 90% of Turkish radiation oncologists surveyed believed that adopting AI would improve their work [29]. While there is optimism regarding the potential of AI, several barriers and concerns might slow the widespread adoption of AI in clinical routine. A key challenge might be represented by the lack of AI expertise. One survey reported that a quarter of radiation oncologists rated their knowledge of AI as “very poor” and 94% expressed a need for further training [29]. The study conducted in New Zealand identified lower familiarity with AI as a barrier, which correlated with a lower intention to use AI.

In addition, a Canadian survey found that while most healthcare professionals recognize the potential of AI to improve patient care, concerns about the threat of job displacement and about changing professional roles contribute to some reluctance [28]. Addressing these psychological barriers is critical; raising awareness of AI as a collaborative tool, rather than a replacement threat, can help foster trust and acceptance. Rosenbacke et al. highlight that so-called explainable AI (XAI), which provides clear, clinically relevant explanations, increased clinicians’ trust. Their findings emphasize the nuanced role of comprehensive explanations [30]. Consequently, one of the most important strategies for driving AI adoption is adequate education and training, as explicitly requested by 94% of surveyed radiation oncologists [29]. Professional workshops and hands-on training may thus help to demystify AI tools. An acceptance study conducted in China reported that clinicians are more likely to adopt AI if they believe it will significantly improve patient care or their workflow efficiency [24]. Successfully integrating AI into radiation oncology requires addressing both technical limitations and human factors. Overcoming skepticism requires a multifaceted approach; education, training, functional transparency, and guided institutional support are all crucial to promote AI adoption. While early adopters pave the way, late adopters can gain confidence as the benefits of AI become increasingly evident in clinical practice.

In our study, AI-based autocontouring was reportedly used in over 90% of cases for OAR contouring of the brain, head and neck, thorax, abdomen, and pelvis, compared to only 43–67% in the UK survey [25], suggesting that AI-based autocontouring is now increasingly used. An overwhelming 88.8% of our participants reported time savings in OAR delineation, with 41.1% estimating savings of 11–20 min per case and 27.1% reporting even greater time savings of over 20 min. These results are comparable to the 88.7% of young French radiation oncologists [20] who reported savings of 25–100% in segmentation time, highlighting the great potential of AI for revolutionizing the time-consuming task of manual segmentation.

While AI-based autocontouring has demonstrated significant time savings in OAR delineation, its application to target volume segmentation remains limited. In our study, only 56.1% of participants had access to an AI solution capable of automatic target volume segmentation. More notably, among those who used such a software, only 40% reported actual time savings in the contouring process. These findings underscore a critical limitation: despite advancements in AI-based autocontouring, its effectiveness and efficiency in target volume segmentation are still lacking. The fact that only about half of the respondents have access to an AI solution for this task—and the fact that the majority of users do not experience meaningful time savings—highlights an unmet need for more reliable and clinically useful AI-driven target volume segmentation tools. Further development and validation of AI models tailored to target volume contouring are necessary to fully harness the potential of automation in radiotherapy planning. Irrespective of these limitations, an overwhelming 92% of all respondents already consider AI-based autocontouring software solutions helpful, underscoring the technology’s perceived value and its promising role in clinical practice.

Given the widespread appreciation of AI-based software benefits, it is not surprising that respondents who added free-text comments highlighted its potential for improving the clinical workflow, addressing staffing shortages, and facilitating the implementation of advanced technologies such as adaptive planning. Others advocated the expansion of AI applications into additional areas of clinical practice. However, while the integration of AI-based autocontouring software has been largely well received, it is important to recognize its limitations and potential risks. Accordingly, respondents raised concerns about quality assurance, education, and training, and warned of the potential deskilling of clinicians and overreliance on automated systems (for more detailed analysis of free-text commentaries, see Appendix B).

To address these challenges, 60% of respondents would welcome guidelines for the implementation and use of AI-based autocontouring software solutions. Indeed, already in 2020, Vandewinckele et al. published recommendations for implementation and quality assurance regarding AI-based applications in radiotherapy [19]. They recommend, as one of our interviewees also noted, the formation of a dedicated multidisciplinary team to ensure safe and appropriate AI use and to educate the entire team on the use and limitations of AI-based autosegmentation. They proposed a two-stage workflow: in the “commissioning phase,” the AI model should be evaluated using an internal dataset. During the “implementation and quality assurance phase”, the implementation team should train and educate all future users in the correct application and interpretation of AI output. Ongoing documentation of the necessary changes, regular meetings between the implementation team and users, and regular quality assurance (QA) of AI output performance following successful implementation have been recommended. Importantly, specific QA runs should specifically address changes in the overall imaging workflow, e.g., after changes in CT scanners or acquisition protocols, as suggested by Vandewinckele et al. In parallel to our study, Hurkmans et al. elaborated “A joint ESTRO and AAPM guideline for development, clinical validation and reporting of artificial intelligence models in radiation therapy” in 2024 [31]. They emphasize the difficulty of validating AI-based segmentation, especially since defining a gold standard or ground truth segmentation is challenging. They also recommend that once an appropriate ground truth has been established, a qualitative (e.g., Likert scale) and a quantitative (e.g., Hausdorff distance [32]) metric should be used as well as a time trial to evaluate the usefulness of the model.

In our view, both reports address in detail aspects relevant to reliably developing and clinically validating AI models, e.g., by implementation of skilled teaching and quality control teams. In light of the very recently published cohesive guideline by the joint European and American expert group [31], the development of a valid and reliable work guide for the implementation of clinically used AI tools, as desired by the majority of our study participants, has already made encouraging progress. We thus further encourage all clinicians already using AI-based autocontouring software solutions to share their experiences and concerns in existing and newly formed national and international expert panels. The thereby-supported continuous improvement of consensus guidelines will then help radiation oncologists considering the implementation of such automated tools in their clinical routine and ensure widespread acceptance and safe implementation of AI-based autocontouring software.

### Limitations

An important limitation of online surveys is response bias: those who favor AI may be more likely to complete the questionnaire. Responses are inherently self-reported and may not reflect the true usage of AI-based autocontouring. The survey was not designed to be representative in terms of providing a complete documentation of the use of AI-based autocontouring solutions in German-speaking radiotherapy clinics and practices, so it is possible that several respondents reflect clustered experiences and the opinions of larger centers. Thus, our study does not provide representative data on the prevalence of actual AI use and acceptance. In addition, topics related to the data security of AI-based autocontouring software solutions were not explicitly addressed in the questionnaire. Cross-professional comparisons are limited by the very unequal numbers of answers from physicists and physicians. Furthermore, although radiation therapists were invited to participate, no DEGRO or ÖGRO representative of this professional group responded, thus further limiting the generalizability of the findings to all relevant professions using AI-based autocontouring software solutions.

## Conclusion

Our survey assessment supports the potential for AI-based autocontouring software to become an integral part of the clinical workflow in radiation oncology. While the majority of respondents are positive about AI, especially concerning the achievable time savings, and see its potential for improving standardization, there is a clear need for ongoing education and thoughtful integration of AI tools into clinical practice. As AI continues to evolve, adaptation of core curricula will be crucial to ensure that AI enhances rather than replaces clinical expertise and skills.

## Appendix

### Appendix A: survey full text

Literal translation from German to English. The original questionnaire was distributed in German.

- 1.1 Which professional group do you belong to? *Drop-down menu.*
  - A: Resident, radiation oncology specialist, physicist, radiation therapist.
- 1.2 How old are you? *Drop-down menu.*
  - A: 20–29 years, 30–39 years, 40–49 years, 50–59 years, ≥ 60 years.
- 1.3 Gender? *Drop-down menu.*
  - A:Female, male, other.
- 1.4 Which country do you work in? *Drop-down menu.*
  - A: Germany, Austria, Switzerland, other (free-text response).
- 1.5 Where do you work? *Drop-down menu.*
  - A: University hospital, non-university hospital, ambulatory health center, medical practice.
- 1.6 Primary contouring of organs at risk (independent of specialist control) is the responsibility of the? *Drop-down menu.*
  - A: Residents, radiation oncology specialists, physicists, radiation therapists.
- 2.1 Do you use AI-based autocontouring software at your workplace? *Drop-down menu.*
  - A: Yes, in clinical routine; Yes, but only for research; No, but implementation is planned within the next year; No, no implementation is currently planned.

Questions 2.2 through 2.11 were asked only of respondents who already use AI-based autocontouring software in routine clinical practice.

- 2.2 Which AI-based autocontouring software do you use? *Drop-down menu.*
  - A: MVision (MVision AI), Limbus AI (Limbus AI Inc.), ART-Plan (TheraPanacea), AI Rad Companion Organs RT (Siemens Healthineers), Ray Station (RaySearch Laboratories), Contour Protégé AI (MIM Software Inc.), Elements (Brainlab AG), other (free text).
- 2.3 How is the AI-based autocontouring software operated? *Drop-down menu.*
  - A: On a local server, cloud-based.
- 2.4 How long have you been using AI-based autocontouring software? *Drop-down menu.*
  - A: < 1 year, 1–3 years, > 3 years.
- 2.5 Did you test different products before purchasing the software solution? *Drop-down menu.*
  - A: Yes, No.
- 2.6 For which OAR do you use AI-based auto-contouring software? *Drop-down menu. Multiple answers possible.*
  - A: Brain, head and neck, thorax, abdomen, pelvic, spine.
- 2.7 How much time do you save on average per contouring by using your autocontouring Software? *Drop-down menu.*
  - A: Extends contouring, None (neither speeds up nor slows down), 0–10 min, 11–20 min, > 20 min.
- 2.8 In how many cases can the autosegmented OARs be adopted without correction? *Drop-down menu.*
  - A: 0–20%, 21–40%, 41–60%, 61–80%, 81–100%.
- 2.9 Do you use automatically generated structures for CTV definition? *Drop-down menu.*
  - A: Not offered by my software solution, No benefit for the contouring time, Saves time during the contouring process.
- 2.10 Is it possible to individualize the CTV definition to the patient’s clinical situation with the available software? *Drop-down menu.*
  - A: Disagree, Rather disagree, Neutral, Rather agree, Agree.
- 2.11 Do the current software solutions reduce the need for staff in your department? *Drop-down menu.*
  - A: Disagree, Rather disagree, Neutral, Rather agree, Agree.
- 3.1 Do you think the use of AI-based autocontouring software is helpful in your clinical practice? *Drop-down menu.*
  - A: Disagree, Rather disagree, Neutral, Rather agree, Agree.
- 3.2 Do you think that the use of AI-based autocontouring software leads to higher standardization and quality assurance? *Drop-down menu.*
  - A: Disagree, Rather disagree, Neutral, Rather agree, Agree.
- 3.3 Do you consider the use of AI-based autocontouring software to be dangerous? *Drop-down menu.*
  - A: Disagree, Rather disagree, Neutral, Rather agree, Agree.
- 3.4 Do you think that using AI-based autocontouring software will degrade resident training in understanding sectional anatomy? *Drop-down menu.*
  - A: Disagree, Rather disagree, Neutral, Rather agree, Agree.
- 3.5 Do you think that the increasing use of AI-based software threatens the existence of radiation oncologists? *Drop-down menu.*
  - A: Disagree Rather disagree, Neutral, Rather agree, Agree.
- 3.6 Would you like to see guidelines for the implementation and use of AI-based autocontouring software by the respective radiation oncology societies? *Drop-down menu.*
  - A: Disagree, Rather disagree, Neutral, Rather agree, Agree.
- 4.0 Do you have any comments on this subject?
  - A: Optional free-text answers.

### Appendix B: analysis of free-text comments

The free-text comments were evaluated and assigned to six different subject areas, whereby a single comment could contain content that could be assigned to several subject areas.

#### (I) Control

Respondents expressed the need for careful oversight of AI-generated structures, emphasizing the importance of manual correction by specialists to ensure accuracy (*n* = 3). Concerns were raised about the potential for errors if AI output is not properly reviewed, especially given the differences in contouring by different specialists. In addition, some highlighted the importance of maintaining standardization and routine corrections to mitigate risk (*n* = 2).

#### (II) Economics

Four respondents highlighted the high cost of AI-based autocontouring software, with some stating that the technology is prohibitively expensive and not yet widely accessible (*n* = 3). Others pointed out that while AI has the potential to improve workflow and reduce overtime work, it has not yet led to significant cost savings or a reduced need for labor.

#### (III) Education and training

Others highlighted the impact of AI on the education and training of medical staff. Concerns were raised about the potential for younger physicians to become overly reliant on AI, leading to a decline in manual skills and critical examination (*n* = 3). Some suggested that AI might require a complete restructuring of assistant training programs to ensure that professionals can identify and correct AI errors. Others noted that training guidelines should be developed to ensure effective use of AI in clinical practice. In addition, the role of radiation therapists in supporting contouring was identified as a potential area for workforce development (*n* = 3).

#### (IV) Implementation

Four respondents emphasized the need for proper implementation strategies when introducing AI into clinical workflows. This includes the creation of dedicated teams of physicians, physicists, and IT specialists with the resources to create best practice protocols for the use of AI-based autocontouring. AI was also seen as essential for addressing staffing shortages and incorporating advanced technologies such as adaptive planning. There were calls for the development of guidelines on how AI should be integrated into routine practice, with some acknowledging that AI is already helping to reduce workload and improve work-life balance.

#### (V) Future outlook

Five respondents were optimistic about the potential of AI, predicting that it might eventually outperform human specialists and provide significant relief as patient numbers continue to grow. The need to fully realize the potential of AI beyond contouring was emphasized, with suggestions to expand its use into different areas of clinical practice. Despite concerns about training, the respondents highlighted AI’s ability to improve quality assurance and standardization. AI was also seen as having a positive impact on the quality of follow-up care and other digital applications. Experienced professionals noted that AI will likely streamline processes in the future, making routine tasks easier and faster.

#### (VI) Limitations

Respondents acknowledged the current limitations of AI (*n* = 5). Some noted that AI still makes serious mistakes and needs to improve significantly before it can be fully relied upon. Concerns were raised that younger professionals might become overly reliant on AI, potentially reducing skill development.
